# An algorithm for discontinuing mechanical ventilation in boys with x-linked myotubular myopathy after positive response to gene therapy: the ASPIRO experience

**DOI:** 10.1186/s12931-024-02966-0

**Published:** 2024-09-16

**Authors:** Robert J. Graham, Reshma Amin, Nadir Demirel, Lisa Edel, Charlotte Lilien, Victoria MacBean, Gerrard F. Rafferty, Hemant Sawnani, Carola Schön, Barbara K. Smith, Faiza Syed, Micaela Sarazen, Suyash Prasad, Salvador Rico, Geovanny F. Perez

**Affiliations:** 1 Boston Children’s Hospital, Harvard Medical School, Boston, MA USA; 2 Hospital for Sick Children, University of Toronto, Toronto, ON Canada; 3 Mayo Clinic, Rochester, MN USA; 4 Great Ormond Street Hospital for Children London, London, UK; 5 MDUK Oxford Neuromuscular Centre, Oxford, UK; 6 Institute I-Motion, Hôpital Armand Trousseau, Paris, France; 7 Brunel University London, London, UK; 8 King’s College London, London, UK; 9 Cincinnati Children’s Hospital Medical Center, Cincinnati, OH USA; 10 University of Cincinnati, Cincinnati College of Medicine, Cincinnati, OH USA; 11 Hauner’s Children’s Hospital, University of Munich, Munich, Germany; 12 University of Florida, Gainesville, FL USA; 13 Formerly of Astellas Gene Therapies, San Francisco, CA USA; 14 Oishei Children’s Hospital, Jacobs School of Medicine and Biomedical Sciences, Oishei Children’s Hospital University at Buffalo, Buffalo, NY USA

**Keywords:** X-linked myotubular myopathy, Neuromuscular disorder, Ventilator independence, Ventilator weaning

## Abstract

X-linked myotubular myopathy (XLMTM) is a rare, life-threatening congenital myopathy. Most (80%) children with XLMTM have profound muscle weakness and hypotonia at birth resulting in severe respiratory insufficiency, the inability to sit up, stand or walk, and early mortality. At birth, 85–90% of children with XLMTM require mechanical ventilation, with more than half requiring invasive ventilator support. Historically, ventilator-dependent children with neuromuscular-derived respiratory failure of this degree and nature, static or progressive, are not expected to achieve complete independence from mechanical ventilator support. In the ASPIRO clinical trial (NCT03199469), participants receiving a single intravenous dose of an investigational gene therapy (resamirigene bilparvovec) started showing significant improvements in daily hours of ventilation support compared with controls by 24 weeks post-dosing, and 16 of 24 dosed participants achieved ventilator independence between 14 and 97 weeks after dosing. At the time, there was no precedent or published guidance for weaning chronically ventilated children with congenital neuromuscular diseases off mechanical ventilation. When the first ASPIRO participants started showing dramatically improved respiratory function, the investigators initiated efforts to safely wean them off ventilator support, in parallel with primary protocol respiratory outcome measures. A group of experts in respiratory care and physiology and management of children with XLMTM developed an algorithm to safely wean children in the ASPIRO trial off mechanical ventilation as their respiratory muscle strength increased. The algorithm developed for this trial provides recommendations for assessing weaning readiness, a stepwise approach to weaning, and monitoring of children during and after the weaning process.

## Introduction

### Weaning off chronic mechanical ventilation

In cases of prolonged respiratory failure, “weaning” describes a gradual, deliberate process aimed at improving the load-to-capacity ratio of the respiratory system’s mechanical and gas exchange capacities to achieve sustainable spontaneous respiration [1, 2]. The most extensive experience for weaning children from chronic invasive or non-invasive respiratory support involves those with chronic lung disease of prematurity [3], congenital malformations (e.g., tracheomalacia, congenital heart disease), and self-limited or acquired neuromuscular conditions (e.g., Guillain-Barré syndrome, spinal cord injury [4–6], critical illness neuromyopathy). Clinical experience in weaning children with neuromuscular disease (NMD) from mechanical ventilation is limited and predominantly focused on extubation within intensive care units (i.e., acute-care settings).

While weaning ventilatory support shares some factors in both adult and pediatric populations, maturational changes such as variations in respiratory mechanics, diaphragm histology, anabolic demands, and developmental tolerance are unique to the pediatric population [7]. Studies on ventilator-induced neuromuscular weakness, particularly diaphragm weakness, in children are limited. Evidence suggests that diaphragm atrophy is associated with prolonged recovery and increased need for noninvasive ventilation (NIV) in acute-care settings [8–12].

In children with chronic lung disease of prematurity, the median age of liberation from respiratory support is 24 months, with variability in clinical practice [13]. However, due to the static or progressive nature of most NMDs, extrapolating from acute-care settings, prematurity, or non-neuromuscular disorders is not necessarily applicable. In fact, published experience in weaning patients with NMDs off mechanical ventilation is limited, except in select cases of congenital myotonic dystrophy [14, 15]. Weaning off transtracheal supports is often not considered feasible from the perspectives of safety or goals of care and frequently requires a transition to noninvasive supportive ventilation [16–19].

### X-linked myotubular myopathy

X-linked myotubular myopathy (XLMTM) is a rare, life-threatening congenital myopathy arising from mutations in the *MTM1* gene, resulting in an absent or dysfunctional myotubularin protein. The disease is characterized in most patients by profound muscle weakness and hypotonia at birth and coinciding severe respiratory insufficiency; absent or transient achievement of motor milestones, including sitting, standing, or walking; and early death [20–23]. At birth, most infants with XLMTM (85–90%) require mechanical ventilation, approximately two-thirds of whom require ventilatory support for > 16 h/day, with some requiring 24-hour ventilation. Most children with XLMTM require permanent invasive respiratory support [22–25] due to severely impaired respiratory mechanics, restrictive disease from thoracic and spinal deformation, and multifactorial cumulative morbidity from dysphagia with subsequent aspiration pneumonitis, impaired lower-airway secretion clearance, and recurrent pneumonia. Ventilator-dependent children with neuromuscular-derived respiratory failure of this nature and degree are not expected to achieve complete independence from mechanical ventilator support [20–22, 24].

An international, observational, run-in study (INCEPTUS, NCT02704273) of 34 children with XLMTM identified baseline ventilator support of 21.4 h/day (SD 4.3) that remained static at 21.7 h/day (SD 4.4) during a median follow up of 13.0 months (range: 0.5, 32.9) [26]. None of the INCEPTUS participants showed significantly improved respiratory mechanics or muscle function, nor did they transition from invasive to non-invasive ventilation [26].

The subsequent ASPIRO clinical trial (NCT03199469) evaluated a single infusion of adeno-associated virus (AAV)-mediated investigational gene therapy (resamirigene bilparvovec, also known as AT132) for the treatment of XLMTM [27]. Upon enrollment, all ASPIRO participants demonstrated severely impaired respiratory muscle function with maximal inspiratory pressures (MIP) four standard deviations below the lower limit of the normal range for age-matched children and required near continuous mechanical ventilator support, averaging 22–23 h/day. The participants, ranging in age from 6.8 to 72.7 months at the time of gene therapy dosing, were primarily cared for in the home setting. By 24 weeks, dosed participants began to show significant improvements in daily hours of ventilation support compared with controls, beyond what investigators had anticipated at study initiation. Eventually, 16 of the 24 ASPIRO participants (67%) who received gene therapy safely discontinued mechanical ventilator support within 14 to 97 weeks after administration [27]. One participant who had been decannulated subsequently required intermittent non-invasive ventilation due to respiratory illness. The ASPIRO trial is no longer screening, enrolling or dosing participants and is now in long-term follow-up for dosed participants. The resamirigene bilparvovec program has been paused to investigate the deaths of four participants who experienced severe cholestatic liver failure after receiving the investigational gene therapy in ASPIRO [27]. The deaths were not related to weaning off ventilatory support.

At the time of the ASPIRO trial, there was no precedent or published clinical guidance for ventilator weaning and/or discontinuing support in this population of children with a rare congenital NMD and chronic ventilator dependence. Consequently, the investigators developed an algorithm to mitigate respiratory complications and facilitate the safe weaning of children from mechanical ventilation. This algorithm aimed to support families and primary respiratory providers, enabling them to potentially pursue tracheal decannulation for those with invasive support, based on their respiratory responses to gene therapy. Here, we present the algorithm developed during the ASPIRO trial, detailing the considerations involved in its development and providing recommendations for monitoring children throughout and following the weaning process.

## Methods

An international group of pulmonologists/respirologists, respiratory physiotherapists, respiratory physiologists, and experts in drug development and managing children with XLMTM (authors RJG, ND, LE, EKF, CL, VM, GFR, CS, BKS, FS, SP, SR, GFP, and contributors CB and CR) was convened in Boston, MA (USA) in April 2018 to devise an algorithm to safely wean ventilatory support for patients in the ASPIRO trial. The algorithm considerations included clinical trial outcomes (i.e., pulmonary function testing and gas exchange) and integrated practical considerations for families and local providers, such as growth, tolerance of intercurrent illnesses, incremental decreases in ventilator support, and observation. This effort was informed by de-identified aggregate data on ventilator dependence, respiratory strength (MIP) data, secretion management, capnography, and polysomnography studies from participants in the ASPIRO trial. The initial algorithm was refined with input from additional experts in the fields of pediatric pulmonology, sleep medicine, respiratory physiology and NMDs (authors RA and HS and contributors MS and AS) and supplemented by a review of literature on weaning in non-neuromuscular conditions and the acute-care setting.

### Algorithm for weaning ASPIRO participants off invasive/transtracheal mechanical ventilation

**Fig. 1:**
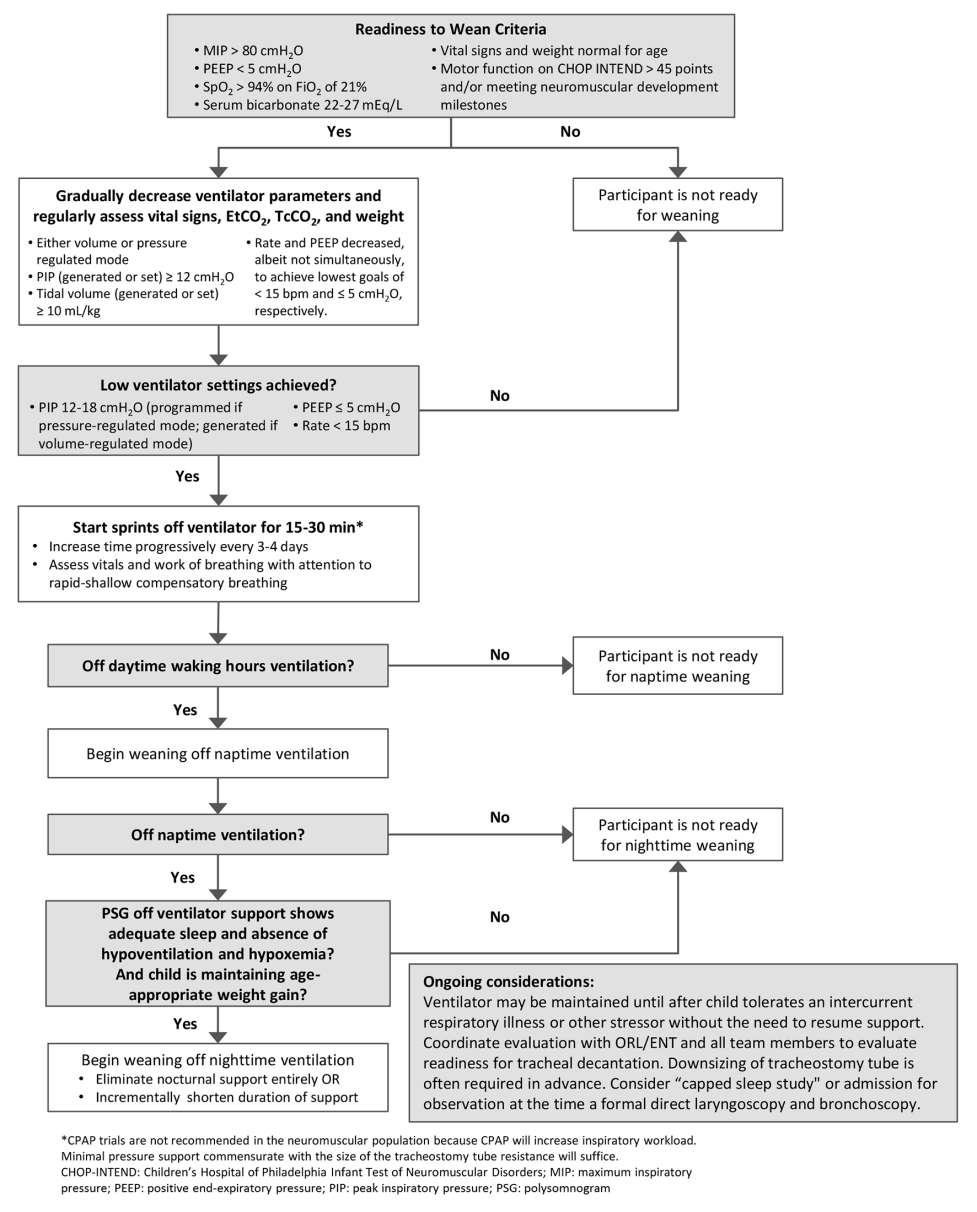
Algorithm for Weaning ASPIRO Dosed Participants Off of Invasive/Transtracheal Mechanical Ventilation

The algorithm in Fig. 1 was developed for the ASPIRO trial and presents a stepwise approach to weaning that involves assessing respiratory function and readiness at each step. Discontinuing mechanical ventilation is a multi-step process, consisting of readiness testing, weaning, and reassessment.

Unlike approaches to other forms of chronic lung disease or acute-care weaning, this guideline was developed in the context of a clinical trial, outlining sequential weans of transtracheal supports and transition to a spontaneous respiratory mode without an anticipated transition to NIV as an intermediate step. While there is experience with continuous NIV in neuromuscular conditions [16–18], the rationale for not transitioning to NIV in this algorithm is multifactorial. Mask interfaces required for NIV may not be tolerated by infants and toddlers unaccustomed to masks [28]. NIV carries potential risk for compromised skin integrity, aspiration, and other aerodigestive considerations [28], and could perpetuate a tracheocutaneous fistula/tract in children with tracheostomy [29], thereby requiring additional surgical intervention. Most importantly, the need for NIV implies ongoing respiratory insufficiency, which supports more conditioning, time, and assessment of capacity to tolerate other stressors (e.g., respiratory infections). Participants in the ASPIRO trial had unprecedented improvement in respiratory muscle function and were deemed capable of discontinuing ventilator support without transitioning to NIV. However, a transition to NIV can be an option for some children, and the authors acknowledge such approaches outside of this clinical trial [30, 31].

#### Determining readiness to wean off mechanical ventilation following investigational gene therapy

Before considering weaning any mechanically ventilated individual, it is essential to establish baselines for airway patency, oxygenation and ventilation capacity, nutritional status, and tolerance of rehabilitation therapies, as well as consider broader patient and environmental factors (Table 1). For participants with XLMTM in the ASPIRO trial, some treatment-emergent adverse events required intensification of immunosuppression, thereby increasing risk of respiratory tract infections in an already at-risk population [32–34]. It is anticipated that similar considerations would apply to future gene therapies in other NMDs.

**Table 1:** Factors to consider before deciding to Wean off Mechanical Ventilation

• Respiratory function (efficacy, efficiency, strength and endurance) • Nature of respiratory failure/insufficiency • Respiratory function while awake vs. asleep • Restrictive respiratory mechanics from long-standing myopathic changes that warrant ongoing supports (e.g., scoliosis) • Secretion management (i.e., oro- and nasopharyngeal as well as tracheal) • Swallow studies • Tolerance of intercurrent respiratory tract infection without needing ventilator support • Health-related quality of life • Chronic lung disease or comorbidities independent of respiratory muscle function	• Cardiopulmonary interactions (i.e., rule-out primary or secondary pulmonary hypertension) • Upper airway assessment (i.e., enlarged tonsils/adenoids, laryngeal and/or tracheomalacia) • Tracheobronchial assessment (i.e., mucosal integrity, laryngeal clefts, tracheomalacia, granulomas, or stenosis) • Nutrition and metabolic demands • Developmental status • Tolerance of interventions (invasive or noninvasive) or need for other adjuvants • Environmental factors (i.e., caution weaning during high-infection seasons) • Immunization status (i.e., fully immunized) • Planned surgeries in the near future

Parameters and values that indicate readiness for reduction in mechanical ventilation support are shown in Table 2. These include respiratory function tests, gas exchange markers, airway patency indicators, nocturnal respiration parameters, polysomnogram results, and clinical judgement. Table 2 also provides guidance on monitoring of children during the weaning process and after the child has successfully discontinued mechanical ventilation. For context, the response to gene therapy in future trials for the NMD population is unknown. However, based on the experience from the ASPIRO trial, frequent assessments are recommended to determine weaning readiness and to guide the reduction of ventilator support. These assessments should be comprehensive with consideration of the parameters and values outlined in Table 2, ensuring any reduction in mechanical ventilation support is safe, well-tolerated, and effective.

**Table 2:** Recommended parameters to evaluate before initiation of Ventilator Weaning based on the ASPIRO experience

Assessments	Parameters
Vital signs	Normal for age
Weight	Normal for age
Respiratory function indicators and measurements	
Maximal inspiratory pressure (MIP)	> 80 cmH_2_O
Maximal expiratory pressure (MEP)	> 40 cmH_2_O
Positive end-expiratory pressure (PEEP) requirement on ventilator	≤ 5 cmH2O
Room air oxygen saturation (SpO_2_)	> 94%
End Tidal CO_2_ (ETCO_2_)	35–45 mmHg
Indirect gas exchange markers	
Serum bicarbonate	22–27 mEq/L
Clinical judgement	Consider week-to-week clinical improvements in motor milestones (rolling over, head control, sitting unassisted); vocalization; coughing; secretions, and neuromuscular function tests (CHOP INTEND)

In the ASPIRO trial, these parameters were used to evaluate the participant’s readiness to wean from positive pressure ventilation. These criteria are evaluated during the in-clinic assessment

#### Gradually decreasing ventilator parameters

Reduction of ventilator settings will depend on the mode of mechanical ventilatory support. The basic approach is to decrease settings progressively and monitor for adequate gas exchange and vital signs. Weaning can be achieved by decreasing either the peak inspiratory pressure (PIP), tidal volume or rate. A gradual decrease in mandatory breaths (i.e., weaning of mandatory breath rate) during the daytime would be considered prior to attempting spontaneous breathing trials off mechanical ventilation. In general, no more than one parameter should be weaned at the same time to avoid weaning failure due to excessive respiratory muscle overload. This will improve recognition of and facilitate interpretation of the responses to different parameters of the weaning process.

The adjustment of ventilator settings during weaning should be tailored to each child, and the child’s overall wellness in response to ventilator changes should be carefully assessed. In general, while working on daytime weaning, the nighttime ventilator settings should be maintained to provide effective recruitment and gas exchange for recovery to maximize respiratory muscle work performance during the day.

Minimum ventilator settings in the range of PIP 12–18 cmH_2_O and peak end-expiratory pressure (PEEP) 4–5 cmH2O are prudent prior to daytime “sprints.” Generated tidal volumes should be continuously monitored, as a decline in volume may indicate underventilation associated with impaired respiratory mechanics or muscle weakness, potentially leading to atelectasis and hypoxemia. In the absence of central neurologic issues, a spontaneous mode of ventilation is preferable. Some providers may opt to maintain a low mandatory respiratory rate of 5–10 breaths per minute overnight, either using a conventional ventilation mode or a hybrid support (e.g., average volume-assured pressure support), with discontinuation of the respiratory rate during the day being an acceptable practice. While weaning ventilator settings during the day, it is important to reduce the fraction of inspired oxygen (FiO_2_) to ambient air and ensure there is no hypoxemia during the weaning period, as hypoxemia can indicate underventilation.

#### Daytime sprinting off ventilator

Once low daytime ventilator settings are achieved, the child’s baseline tolerance for spontaneous breathing can be determined by testing sprint duration in the clinic and progressively adding time off the ventilator from there. Initiation of sprinting should be considered in conjunction with respiratory care providers. The initial sprint trial should be at an office visit with an open tracheostomy or heat moisture exchange for 30 min. Upon successful completion of the in-office trial, sprinting should continue in the homecare setting with established safety parameters (i.e., stopping the sprint if oxygen saturation decreases to < 94%, respiration rate increases more than 25% above baseline, ETCO_2_ increases to > 50 mmHg, or compensatory tachycardia is observed). For example, 15-minute sprints, two times per day, and then increasing each of the sprints by 15 min until ventilator support is only on for naps during the day. (e.g., 1 h, then 2 h, then 3 h and so on).

Providers can assist families in determining which daytime weaning schema is preferable—single sessions of longer duration or multiple “sprints” of shorter duration—until these sessions merge. From a neuromuscular perspective, the latter has implicit benefit with muscle conditioning and interval rests in patients with otherwise improving muscle strength and function. The frequency of these incremental changes may vary between individuals and, potentially, for a given child based upon the trajectory of response to gene replacement therapy or other stressors; tailoring to the individual may allow for home-based extension weekly or more frequently.

#### Discontinuing daytime mechanical ventilation

The respiratory assessments in Table 2 should be performed several times prior to attempting ventilator weaning. Children who meet the readiness criteria outlined in Table 2 can move forward with daytime weaning. These recommended values are proposed in the context of the ASPIRO clinical trial (reported separately) [27]. They are intended to be more conservative, recognizing the uncertainty of the clinical trial and the intuitive need to optimize respiratory support with dynamic metabolic and catabolic demands in response to the investigational gene therapy. Weaning usually involves a gradual reduction in ventilator support (i.e., pressure/volume/rate) for patients on higher settings and continuous support, followed by progressive sprinting off the ventilator [35, 36]. Upon demonstrating capacity and appropriate airway manometry, a speaking valve or cap could be utilized under direct observation [37].

Throughout the weaning process, providers and parents should be attentive to the work of breathing, compensatory tachypnea, compensatory tachycardia, and other clinical evidence of distress [1]. Assessment of spontaneous secretion clearance or, conversely, the need for additional cough augmentation or suctioning should also guide the process. A pulse oximeter should be used at all times to monitor oxygen saturation and heart rate. Stopping the daytime weaning process should be considered if oxygen saturation decreases to < 94% or by 3–4% from baseline or if heart rate increases more than 20 bpm from baseline (baseline is defined as the time of weaning assessment with reassessment at subsequent trial encounters). Tachycardia can be an indicator of cardiac compensation for respiratory insufficiency, hypoventilation, or for indolent hypercapnia prior to oxygen desaturation [38]. It is important to note that children with neuromuscular weakness may not show the typical signs of respiratory failure, such as retractions and compensatory tachypnea; thus, other signs including tachycardia and behavioral/emotional cues should prompt the clinician to stop the weaning process.

#### Weaning off naptime ventilation

When the child has successfully discontinued ventilator support during waking daytime hours, consider initiating the process of weaning off ventilator support during naptimes to assess respiratory efficiency during sleep. A pulse oximeter should also be used during naptimes to monitor for oxygen desaturation and heart rate increases; the latter may be a surrogate for cardiorespiratory compensation or indolent hypercapnia prior to desaturation. If available, a home-based end-tidal (ET) or transcutaneous CO_2_ can be beneficial, though it is possible that variable availability and experience using these monitors may present challenges [39]. Consider stopping the naptime weaning process if there is tachypnea, oxygen saturation decreases to < 94% or by 3–4% from baseline, heart rate increases more than 20 bpm from baseline, or ETCO_2_ or transcutaneous CO_2_ is above 50 mmHg or increases 10 mmHg above the awake baseline [40].

#### Weaning off nighttime ventilation

When the child has successfully discontinued ventilator support during both waking and napping daytime hours, begin the process of discontinuing nighttime ventilator support onto an open trach/trach collar. Before eliminating nighttime ventilator support, a polysomnogram should be conducted as it is the gold standard for assessing sleep-disordered breathing in patients with NMD [41]. The polysomnogram should be performed off the ventilator with the tracheostomy open for invasively ventilated children or with the mask off for noninvasively ventilated children. If a polysomnogram is not feasible, the best surrogate is nocturnal capnometry (i.e., using a digital monitoring system) in patients with access to this technology, for 2–3 nights off the ventilator before discontinuing the ventilator entirely.

Once a polysomnogram shows stable oxygenation and ventilation and acceptable sleep quality with an open tracheostomy, nighttime weaning can be accomplished either by reducing the number of ventilator support hours per night at regular intervals or by eliminating nocturnal support entirely. Eliminating nocturnal support in a single step avoids the burden of lost sleep associated with weaning at hourly increments. The need for “nocturnal conditioning” also suggests that the child may not be ready for discontinuation and might require resumption of respiratory support with any stressor. The nocturnal respiration monitoring and polysomnogram parameters recommended for discontinuation of mechanical ventilation are shown in Table 3. Nocturnal monitoring of oximetry, heart rate, estimates of respiration rate, and, if available, home ETCO_2_ monitoring should be continued after nighttime ventilator support is discontinued for the first 6–8 weeks. Alternatively, the child could be admitted to the hospital for nighttime close monitoring and blood gases in the morning. A follow-up polysomnogram is recommended for children who have changes in clinical course, including mild desaturations, poor weight gain, or mood changes. Thereafter, correlation of gross motor trajectory with standardized neuromuscular measures (e.g., Children’s Hospital of Philadelphia Infant Test of Neuromuscular Disorders [42], Motor Function Measure-20 short scale [43], or Bayley Scales of Infant and Toddler Development 4th edition [44] gross motor function domain) and/or tracking attainment of major motor milestones (e.g. sitting unassisted for > 30 s, standing, walking with and without support), and general clinical status can inform the need for follow-up polysomnogram. Plateau or loss of gross motor function may correlate with similar respiratory muscle function or portend subacute respiratory events.

**Table 3:** Recommended parameters for Guiding Discontinuation of Mechanical Ventilation after positive response to Gene Therapy based on the ASPIRO experience

Assessments	Parameters
Gas exchange markers	
Sprinting (video record the assessment)	No distress or SpO_2_ < 94% or TCO_2_ > 45 mmHg during respiratory sprinting trial No intercostal retraction, tachypnea, or respiratory paradox
**Nocturnal respiration monitoring**	
Transcutaneous CO_2_ (TcCO_2_)	35-45mmHg
End Tidal CO_2_ (ETCO_2_)	35-45mmHg
Oxygen saturation (SpO_2_)	> 94%
Respiration rate (RR)	Within the age adjusted norms
**Polysomnogram (PSG)** (performed with trach open)	
Apnea-hypopnea index (AHI)	< 5 events/hour (lower threshold for NMD than for obstructive processes)[40]
TcCO_2_	35–45 mmHg
PetCO_2_	< 50 mmHg or sleep-related or increase < 10 mmHg above the awake baseline[40]
Partial pressure of oxygen (PO_2_)	Not typically measured
Oxygen saturation (SpO_2_)	> 94%[40]
Respiration rate (RR)	Within the age adjusted norms

### Ongoing considerations

#### Assessing weaning outcomes

With any weaning strategy, the clinician must determine whether weaning was a success or a failure. Objective criteria that may indicate weaning failure include tachypnea, respiratory distress (use of accessory muscles, thoracoabdominal paradox, and diaphoresis), hemodynamic changes (tachycardia, hypertension), oxygen desaturation, hypercapnia, and changes in mental status (somnolence, agitation, or more subtle behavioral changes). In addition, parents and providers should continue to be attentive to and report daytime and/or longer-term symptomology, such as fatigue, headache, failure to thrive (weight loss or slowing of growth), and/or intolerance of activities and physical therapy.

Upon cessation of invasive ventilator support and after monitoring the child adequately for 6–12 months, encompassing assessments during respiratory infections, a tracheostomy decannulation process can be started. This decision stems from the established adequacy of respiratory muscle function, including bulbar muscle function, alongside successful weaning from respiratory support. Utilization of standard decannulation protocols is appropriate. These protocols typically involve a step-by-step progression including implementation of a speaking valve, use of a tracheostomy cap, airway endoscopy, and comprehensive respiratory monitoring, which may include polysomnography or in-hospital monitoring [45–48].

#### Secretion management and/or intercurrent illness or infection

Augmented secretion clearance may facilitate ventilator weaning and sprinting periods. As such, a detailed, individualized, multidisciplinary secretion management plan should be established for the periods before, during, and after weaning based on the child’s overall health. Any combination of chest physiotherapy, mechanical cough assistance, tracheal suctioning, passive lung recruitment, and/or manual bag breaths (i.e., air stacking) may be advisable before a sprinting trial to minimize airway obstruction and atelectasis at the outset. However, the increased need for airway clearance intervention during a sprint may suggest the need to resume ventilator support, as it is an indicator of insufficient respiratory capacity.

Weaning should be paused during any illness with increased respiratory and metabolic demands, and capacity should be reassessed following recovery. Specifically, if the child is off ventilator support but still has a tracheostomy, providers should consider resumption of such ventilator support if the need for supplemental oxygen or respiratory distress are identified. An increase in frequency or severity of illness during the weaning period may be indicative of an increased need for respiratory support.

There is also an appreciation that children who have sustained lung injury from chronic aspiration or recurrent pneumonia may have limited potential to wean off respiratory support. It may be necessary to initiate a parallel approach to chronic parenchymal lung disease (i.e., non-CF bronchiectasis, fibrosis) along with the NMD guidelines as well as airway considerations [47]. In this instance, mechanical ventilation may not necessarily be a long-term need; supplementary oxygen may be the only requirement in children with adequate ventilation and respiratory muscle function. However, clinicians should heed the warning of hypoxemia as it can reflect ventilation-perfusion mismatching and oxygen supplementation can mask hypoventilation.

#### Weight maintenance

Failure to thrive during weaning in the absence of other factors may imply that the caloric demands of weaning and spontaneous breathing are exceeding caloric intake. The answer may not be an empirical increase in calories, as this may increase the CO_2_ burden and consequently exacerbate hypoventilation. Close monitoring by an experienced team is warranted.

#### Daytime activity

Close attention to tiredness is important, and ensuring adequate rest is essential. If spontaneous activity declines during weaning, then the ventilator may be utilized to aid in recovery or to revert to the prior level of support. Signs of fatigue, which may manifest as reduced endurance in routine physical therapy sessions, may indicate that weaning is proceeding too quickly. Ideally, the child should be able to maintain his/her prior activity levels during weaning.

#### Interventions in the presence of fixed-restrictive lung disease

The need for spinal instrumentation to address neuromuscular scoliosis (growth rods, vertical expandable prosthetic titanium rib, etc.) may necessitate prolonged post-operative respiratory support that would be facilitated by a tracheostomy and a ventilator or noninvasive ventilation. For patients whose thoracic cage is rigid and restrictive, there may be a degree of persistent respiratory insufficiency that is not recoverable, independent of muscle strength [49]. This may prompt discussions about longer-term options and would be informed by polysomnograms and other clinical indicators, as delineated above.

## Conclusions

Clinical care for children with NMDs varies across providers and institutions worldwide, with variable interpretation of, and adherence to [50–52], established respiratory care guidelines [38, 53–55]. The algorithm and guidelines outlined here acknowledge differences in existing care regimens, while highlighting developmentally appropriate pathways and creating clinical boundaries to direct, and potentially limit, the weaning process in the context of a gene-replacement therapy clinical trial. A multidisciplinary approach is essential to ensure that clinical teams, research teams, primary investigators, and the pulmonary/respiratory rehabilitation team are closely aligned and regularly share data and care information. It is also important to communicate with the child’s other health care providers, including physical therapist, speech pathologist, and nutritionist, to determine therapy modifications based on the child’s improvements.

The opportunity for ventilator-dependent children with XLMTM to breathe without ventilator assistance is a novel development. Close follow-up and regular respiratory assessments will be needed given the unknown trajectory and durability of respiratory outcomes after weaning these patients off mechanical ventilation. While this algorithm was developed and utilized in an XLMTM population during the ASPIRO clinical trial of an investigational gene therapy, it can serve as a frame of reference for other clinical trials in children with NMDs.

## Data Availability

No datasets were generated or analysed during the current study.

## Abbreviations

AAV: Adeno-Associated Virus
NIV: Noninvasive Ventilation
NMD: Neuromuscular Disease
PEEP: Positive End-Expiratory Pressure
PIP: Peak Inspiratory Pressure
XLMTM: X-linked Myotubular Myopathy
